# Supplementary material for: Real-World Data of Trastuzumab Deruxtecan for Advanced Gastric Cancer: A Multi-Institutional Retrospective Study
Source: J Clin Med. 2022 Apr 17;11(8):2247. doi: 10.3390/jcm11082247 (PMC9030612; doi:10.3390/jcm11082247)
Supplement: Supplementary file 1 [file jcm-11-02247-s001.zip › jcm-1687238-supplementary.pdf]

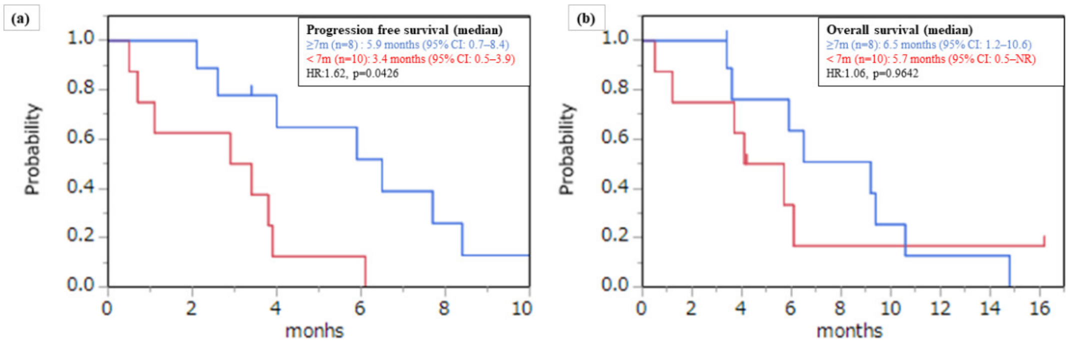

Figure S1. (**a**) PFS and (**b**) OS classified by efficacy of prior Tmab containing regimen. Red line: 7 months > PFS of previous Tmab containing regimen, Blue line: 7 months ≥ PFS of previous Tmab containing regimen.

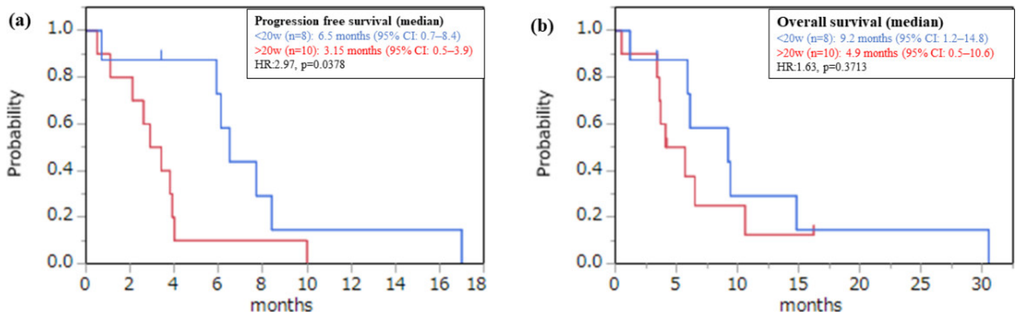

Figure S2. (**a**) PFS and (**b**) OS classified by ICI free interval. Red line: 20 weeks < Immune checkpoint inhibitor (ICI) free interval, Blue line: 20 weeks > ICI free interval.
